# Early newborn bathing and associated factors among mothers in Afar Region, Northeast Ethiopia

**DOI:** 10.1093/tropej/fmac117

**Published:** 2023-01-10

**Authors:** Gebru Getachew, Ibrahim Mohammed Ibrahim, Yisahak Mulugeta, Kedir Y Ahmed

**Affiliations:** Department of Nursing, Semera Health Science College, Samara, Afar, PO Box: 142, Samara, Ethiopia; Department of Midwifery, College of Medical and Health Science, Samara University, PO Box: 132, Samara, Ethiopia; Department of Midwifery, Semera Health Science College, Samara, Afar, PO Box: 142, Samara, Ethiopia; Rural Health Research Institute, Charles Sturt University, Orange, NSW 2800, Australia; Translational Health Research Institute, Western Sydney University, Campbelltown Campus, Locked Bag 1797, Penrith, NSW 2751, Australia

**Keywords:** early newborn bathing, postnatal mothers, pastoral communities, Afar Region, Ethiopia

## Abstract

**Background:**

Delaying newborn bathing for 24 h after childbirth protects the baby from hypothermia, infection and hypoglycaemia and provides an opportunity for mother–baby emotional bonding. However, no previously published study has investigated the early newborn bathing practices of pastoral mothers in Ethiopia. This study aims to investigate early newborn bathing and associated factors among mothers in Afar Region, Northeast Ethiopia.

**Methods:**

Institution-based cross-sectional study was conducted from May to June 2021. A systematic random sampling technique was used to recruit 386 mothers, and the data collection was performed using an interviewer-administered questionnaire. Multivariable logistic regression modelling was used to examine the association between explanatory variables (including sociodemographic, obstetric, health service and health literacy factors) and early newborn bathing.

**Results:**

The overall prevalence of early newborn bathing among postpartum mothers was 73.1% with a 95% confidence interval (CI) from 68.4 to 77.5%. Mothers who attained college or higher education [adjusted odds ratio (AOR) = 0.21; 95% CI 0.06–0.66], those who were from urban areas (AOR = 0.19; 95% CI 0.09–0.42) and those who gave birth using operational delivery (e.g. caesarean section and instrumental delivery) (AOR = 0.01; 95% CI 0.01–0.04) were less likely to practice early newborn bathing.

**Conclusion:**

The practice of early newborn bathing was unacceptably high in pastoral communities of the Afar Region. There is a need for interventions specifically targeting at uneducated and rural mothers as part of the implementation to improve the essential newborn care practices of mothers in pastoral communities in Ethiopia.

## BACKGROUND

Improving the health status and survival of children in the first month of life is essential to achieve the sustainable development goal target 3.2 ending preventable deaths of newborns and children under 5 years of age by 2030 [1]. Evidence from the World Health Organisation and United Nations Children’s Fund (WHO/UNICEF) showed that 2 million newborns died during their first 7 days of life globally and half occur during the first 24 h, and nearly half of all under-5 deaths occur during the first 28 days of life [2, 3]. Sub-Saharan Africa (SSA, including Ethiopia) and central and southern Asia have the highest neonatal mortality rates in the world attributable to 43 and 36% of global newborn deaths, respectively [3]. Also, a child born in SSA is 10 times more likely to die in the first month than a child born in a high-income country [4].

Ethiopia has seen progress in reducing the neonatal mortality rate from 47.9 per 1000 in 2000 to 30.0 per 1000 in 2016 [5–8]. The achievement could potentially be because Ethiopia has implemented several interventions and programmes in the past two decades to improve the health and survival status of mothers and children. For example, the Ethiopian Federal Ministry of Health developed a national strategy for addressing maternal and newborn health with the primary health care approach and health extension package since the 1990s, and the country has also recently designed the newly revised child survival and newborn care strategy 2016–20 [9–12]. The Health Sector Transformation Plan (HSTP-I) was also implemented from 2015 to 2019 with the main aim of improving health outcomes (including maternal and child health outcomes) and increasing access to and utilization of health services [13].

Although these initiatives are important in reducing deaths, in Ethiopia, neonatal mortality is still among the highest in the world, nearly 122 000 babies die every year within the first month of birth [14], and the proportion of neonatal deaths from a total under-5 deaths increased from 43% in 1990 to 56% in 2019 [5–8]. Neonatal hypothermia is one of the main contributing factors for neonatal morbidity and mortality for both low-birth weight and normal-weight babies, even in tropical countries (including Ethiopia) [15]. Essential thermal care practices such as immediate drying and wrapping, skin-to-skin contact, early initiation of breastfeeding and delaying bathing until the second day of life are the recommended interventions for reducing the early death of newborns attributed to hypothermia [15, 16].

Delaying newborn bathing for 24 h preserves the body heat with a positive impact on the infant’s temperature regulation and glucose stability and promotes initiation of breastfeeding, emotional bonding and skin-to-skin care [17]. However, prior studies conducted in Ethiopia [18, 19] showed many women are still bathing their babies earlier than 24 h postpartum. Spontaneous vaginal birth [19], low maternal education [18, 19], poor knowledge of obstetric danger signs and hypothermia [18, 19] and young mothers [18] were the common factors positively associated with early newborn bathing in Ethiopia. Investigating the magnitude and associated factors of early newborn bathing would be important for pastoral communities (including Afar Region) to formulate specific interventions on drivers of early newborn bathing. The study aimed to investigate the prevalence of early newborn bathing and associated factors in the Awsi Rasu Zone of the Afar National and Regional State, Northeast Ethiopia.

## MATERIALS AND METHODS

### Study design and study setting

**Figure 1:**
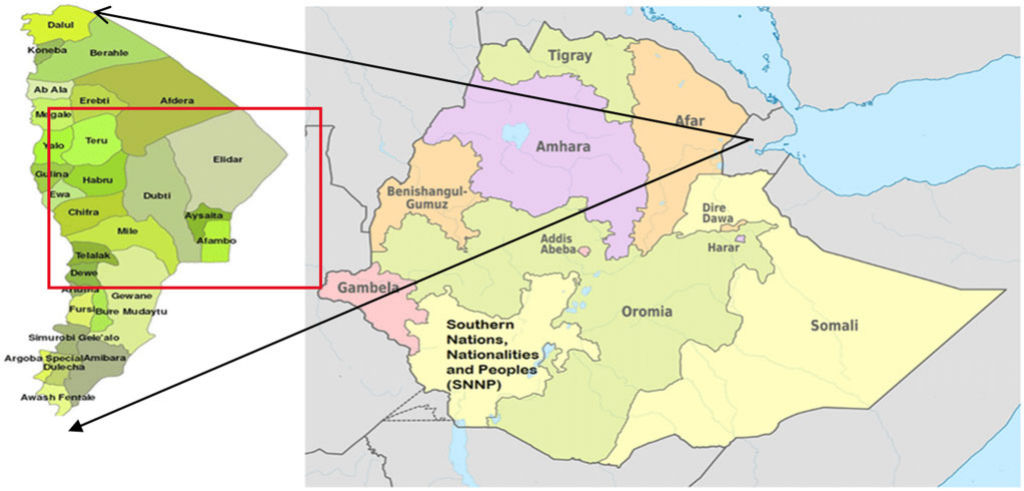
The administrative map of the Afar National Regional State in Ethiopia.

An institution-based cross-sectional study design was conducted to investigate the prevalence of early newborn bathing and associated factors among mothers who visited the Expanded Program of Immunization (EPI) clinic in public health facilities of the Awsi Rasu Zone of the Afar Region from May to June 2021. The Afar National Regional State is geographically located in the Great East African Rift Valley, shares a border with Eritrea (Northeast), Tigray (Northwest), Oromia (South), Somali (Southeast), Amhara (West) and Djibouti (East) [20]. The ANRS has an estimated population size of almost 2 million with more than 12% of the population aged less than 5 years, and the livelihood of more than 85% of the Afar population depends on livestock production (including camel, goat and cow) [21]. The arid or semi-arid and low rainfall climate makes the ANRS vulnerable to adverse health problems including malnutrition, infection and poor maternal and child survival [20]. The Awsi Rasu Zone has three public hospitals, one private maternity hospital and 25 health centres that provide preventive, curative and rehabilitative health services (Figure 1).

### Source and study population

All postpartum mothers who visited EPI clinics of public health facilities in the Awsi Rasu Zone of the Afar Region were the source population, and those randomly selected mothers were the study population. Mothers who were seriously sick during the data collection period and unable to hear were excluded.

### Sample size determination and sampling procedure

A total of 386 postpartum mothers were selected using a single population proportion formula, assuming a proportion of 35.4% (based on a study from Eastern Ethiopia) [18], 95% confidence interval (CI), 5% margin of error and 10% non-response rate. Dubti Referral Hospital and five randomly selected health centres (Chifra Health Centre, Samara Health Centre, Logia Health Centre, Magenta Health Centre and Mille Health Centre) were included in the study. Postpartum mothers in the selected health facilities were selected using a systematic random sampling method proportional to the patient flow in the EPI clinic of health facilities.

### Outcome variable

The main outcome variable of the study was time to early newborn bathing dichotomized as (‘1 = Yes for early newborn bathing’ or ‘0 = No for not early newborn bathing’), consistent with the WHO recommendation and previously published studies [18, 19, 22, 23].

### Independent variables

We broadly grouped independent variables into socio-demographic, obstetric, health service and health literacy factors. Sociodemographic factors included maternal age (grouped as ‘15–24 years’, ‘25–34 years’ or ‘35–49 years’), mother’s education (grouped as ‘nor formal schooling’, ‘primary schooling’, ‘high school and preparatory’ or ‘college or higher education’), mother’s occupation (grouped as ‘housewife’, ‘pastoralist’ or ‘government or private employed’) and father’s occupation (grouped as ‘pastoralist’, ‘government or private employed’ or ‘merchant’).

Obstetric and health service factors included antenatal care (ANC) use (grouped as ‘Yes’ or ‘No’), ANC provider (grouped as ‘Doctor’ or ‘Midwifery’), ANC counselling (grouped as ‘Yes’ or ‘No’), number of pregnancies (grouped as ‘1–2 pregnancies’, ‘3–4 pregnancies’ or ‘5+ pregnancies’), postnatal care (PNC) use (grouped as ‘Yes’ or ‘No’), place of birth (grouped as 'home’, ‘clinics’, ‘health centre’ or ‘hospital’), mode of delivery (grouped as ‘standard vaginal delivery’ or ‘operational delivery’) and delivery assistance (grouped as ‘physician’, ‘other health care workers’ or ‘traditional birth attendants’). We also measured health literacy factors such as awareness of the danger signs of a newborn (grouped as ‘Yes’ or ‘No’) and exposure to media outlets, including radio, magazine and television (grouped as ‘Yes’ or ‘No’).

### Data collection

We used an interviewer-administered questionnaire to collect sociodemographic, obstetric, health service and health literacy factors, and time to newborn bathing. The questionnaire was first prepared in English and translated to the local language (Amharic) and translated back to English to check for consistency. Data collectors were from the local community and spokes both Amharic and Afar (Qafar’af) languages. The data collection was performed by four trained diploma nurses (who were not working in the study area), and bachelor of midwifery professionals supervised the data collection process. The content of the questionnaire was prepared based on previously published studies in Ethiopia [18, 19].

### Data quality control

We provided an appropriate training for the data collectors and supervisors, and a pre-test was conducted on 19 postpartum mothers in Aysaita Primary Hospital (not selected for the current study), and appropriate amendments were made based on the findings of the pre-test. During the data collection, a daily meeting was conducted among the principal investigator, supervisors and data collectors to check the completeness and clarity of the completed questionnaire and to solve unanticipated problems promptly.

### Data management and statistical analysis

Data were cleaned, coded and entered into Epi info version 4.2, and exported to Statistical Package for Social Science (SPSS) version 25 for the final analysis (IBM Corp. [24]). Multivariable logistic regression modelling was used to investigate the association between sociodemographic, obstetric, health service and health literacy factors with the main outcome (early newborn bathing). Frequencies, percentages, mean and standard deviation were used to describe the study participants. Odds ratios (ORs) and 95% CI were used to report the findings from the regression modelling, and *p*-value <0.05 was used to declare statistical significance.

### Ethical consideration and consent to participate

Ethical approval was obtained from the Research Ethics Review Committee of Samara University, College of Medicine and Sciences. Formal permission letters were also obtained from the Afar Regional Health Bureau and the respective health facilities. Informed verbal consent was obtained from mothers after explaining the purpose of the study, the importance of their participation and the right to withdraw from the study at any time. Privacy and confidentiality of the information obtained from each respondent were kept properly and the name was not recorded, and the recorded data were kept in a locked cabinet with security.

## RESULTS

### Characteristics of respondents

Data were collected from 383 study participants with a response rate of 99.2%. Half of the mothers were in the age group 25–35 years, and 54% of them did not have formal education. Most mothers were housewives (76.2%), and 36.0% of fathers were government employed. Half of the study participants were rural residents (50.4%) (Table 1). One in three mothers reported three ANC visits for the current child (65.0%), and 67.4% of mothers reported delivery assistance by a skilled health provider. About 15.7% of mothers heard newborn danger signs before, and only 2.9% of mothers listened to the radio. More than one-third of mothers reported watching television (Table 2). Among respondents, more than half 53.5% of them gave birth during Autumn (March–May), and the majority of them initiated breastfeeding within the first hour of childbirth (93.0%). More than one-third of mothers applied local substances to the umbilical cord during childbirth (36.8%) (Table 3).

**Table 1.** Sociodemographic characteristics of study participants in the Awsi Rasu Zone of Afar Region, Northeast Ethiopia (*N* = 383)

Variables	Frequency (*n*)	Percentage (%)
Mother’s age (years)		
15–24	183	47.8
25–35	192	50.1
35–49	8	2.1
Place of residence		
Rural	193	50.4
Urban	190	49.6
Religion		
Muslim	289	75.5
Orthodox	94	24.5
Ethnicity		
Afar	180	47.0
Amhara	130	33.9
Tigre	45	11.7
Oromo	28	7.3
Marital status		
Married	377	98.4
Cohabiting	6	1.6
Mother’s education		
No formal education	207	54.0
Primary school	82	21.4
High school or preparatory	59	15.4
College or higher	35	9.1
Mother’s occupation		
Housewife	292	76.2
Pastoralist	44	11.5
Government employed	38	9.9
Self-employed	9	2.3
Father’s occupation		
Pastoralist	49	12.8
Self-employed	124	32.4
Merchant	72	18.8
Government employed	138	36.0

**Table 2.** Obstetric, health service and health literacy factors in the Awsi Rasu Zone of Afar Region, Northeast Ethiopia (*N* = 383)

Variables	Frequency (*n*)	Percentage (%)
ANC follow-up		
Yes	343	89.6
No	40	10.4
Frequency of ANC visits (*n* = 343)		
One visit	2	0.6
Two visits	34	9.9
Three visits	223	65.0
Four and above visits	84	24.5
ANC providers		
Doctors	89	25.9
Midwifery	254	74.1
Received ANC counselling		
Yes	316	92.1
No	27	7.9
Number of pregnancies		
≤2 pregnancies	213	55.6
3–4 pregnancies	147	30.4
5+ pregnancies	23	14.0
Place of birth		
Home	41	10.7
Private health facility	58	15.1
Health centre	106	27.7
Government hospital	178	46.5
Mode of delivery		
Normal vaginal delivery	334	87.2
Operational delivery	49	12.8
Delivery assistance		
Physician	83	21.6
Other health care provider	258	67.4
Traditional birth attendant	42	11.0
Knowledge of newborn danger sign		
Yes	60	15.7
No	323	83.3
Newborn danger signs reported (*N* = 60)		
Unable to breastfeed	54	14.1
Fast breathing	53	13.9
Chest indrawing	35	9.1
Fever	59	15.4
Listening radio		
Yes	11	2.9
No	372	97.1
Watching television		
Yes	289	75.5
No	94	24.5

**Table 3.** Newborn care practices in the Awsi Rasu Zone of Afar Region, Northeast Ethiopia (*N* = 383)

Variables	Frequency (*n*)	Percentage (%)
Presence of vernix caseosa at birth		
Yes	53	13.8
No	330	86.2
Season of current baby birth		
Summer (December–February)	178	46.5
Autumn (March–May)	205	53.5
Substance on umbilical cord		
Yes	141	36.8
No	242	63.2
Breastfeeding initiation		
≤1 h	356	93.0
>1 h	27	7.0
Exclusive breastfeeding		
Yes	199	52.0
No	184	48.0

### Prevalence of newborn bathing

**Figure 2:**
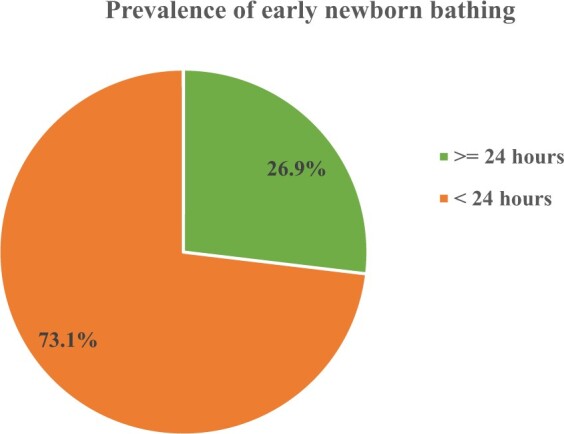
Prevalence of early newborn bathing in the Awsi Rasu Zone of Afar Region, Northeast Ethiopia (*N* = 383).

The overall prevalence of early newborn bathing among postpartum mothers was 73.1% with a 95% CI from 68.4 to 77.5% (Figure 2). Rural residents had a higher prevalence of early newborn bathing compared to urban residents (88.6 vs. 57.3%), and mothers who did not have formal education had a higher prevalence of early newborn bathing compared to those with college or higher education (83.0 vs. 42.8%). Government or privately employed mothers had a lower prevalence of early newborn bathing compared to pastoralist mothers (55.3 vs. 84.1%), while mothers who did not visit health facilities for ANC had a higher prevalence of early newborn bathing compared to those who had four or more ANC visits (87.5 vs. 54.8%) (Table 4).

**Table 4.** Factors associated with early newborn bathing among mothers in the Awsi Rasu Zone of Afar Region, Northeast Ethiopia (*N* = 383)

Variables	Early newborn bathing		COR (95% CI)	AOR (95% CI)	*p*-value
	Yes, *n* (%)	No, *n* (%)			
Place of residence					
Rural	171 (88.6)	22 (11.3)	1.00	1.00	
Urban	109 (57.3)	81 (42.6)	0.17 (0.10–0.29)	0.19 (0.09–0.42)	<0.001
Mother’s age (years)					
15–24	132 (72.1)	51 (27.8)	1.00	1.00	
25–34	143 (74.5)	49 (25.5)	1.13 (0.71–1.78)	2.65 (0.59–11.4)	0.203
35+ years	5 (62.5)	3 (37.5)	0.64 (0.14–2.79)	2.27 (0.39–13.2)	0.362
Mother’s education					
No formal schooling	172 (83.0)	35 (16.7)	1.00	1.00	
Primary school	54 (65.8)	28 (34.1)	0.39 (0.22–0.70)	0.54 (0.20–1.05)	0.065
High school or preparatory	39 (66.1)	20 (33.8)	0.40 (0.21–0.76)	0.41 (0.17–1.03)	0.057
College or higher	15 (42.8)	20 (57.1)	0.15 (0.07–0.33)	0.23 (0.75–0.71)	0.010
Mother’s occupation					
Pastoralist	37 (84.1)	7 (15.9)	1.00	1.00	
Housewife	217 (74.3)	75 (25.7)	0.55 (0.23–1.28)	2.30 (0.41–12.91)	0.345
Employed	26 (55.3)	21 (44.7)	0.23 (0.09–0.63)	1.89 (0.27–13.41)	0.520
Father’s occupation					
Pastoralist	40 (81.6)	9 (18.4)	1.00	1.00	
Employed	190 (72.5)	72 (27.5)	0.59 (0.27–1.29)	1.62 (0.33–8.08)	0.552
Merchant	50 (69.4)	22 (30.6)	0.51 (0.21–1.23)	1.12 (0.20–6.26)	0.894
Number of pregnancies					
≤2 pregnancies	152 (71.4)	61 (28.6)	1.00	1.00	
3–4 pregnancies	112 (76.2)	35 (23.8)	1.28 (0.79–2.08)	1.48 (0.76–2.89)	0.244
5+ pregnancies	16 (69.6)	7 (30.4)	0.92 (0.36–2.34)	1.14 (0.32–4.13)	0.839
ANC visits					
None	35 (87.5)	5 (12.5)	1.00	1.00	
1–3 visits	199 (76.8)	60 (23.2)	0.47 (0.18–1.26)	0.43 (0.07–2.42)	0.335
4+ visits	46 (54.8)	38 (45.2)	0.17 (0.06–0.48)	0.24 (0.04–1.44)	0.119
Mode of birth					
SVD	274 (82.0)	60 (18.0)	1.00	1.00	
Operational delivery	5 (10.4)	43 (89.6)	0.03 (0.01–0.07)	0.01 (0.003–0.04)	<0.001
Watching television					
No	81 (84.4)	15 (15.6)	1.00	1.00	
Yes	199 (69.3)	88 (30.7)	0.42 (0.23–0.77)	0.97 (0.30–3.17)	0.965
Early initiation of breastfeeding					
No	11 (40.7)	16 (59.3)	1.00	1.00	
Yes	269 (75.6)	87 (24.4)	4.50 (2.01–10.06)	0.83 (0.20–3.47)	0.801

NB, In the Ethiopian context, primary school represents 1–8 grades and high school represents 9–12 grades; employed represents both government and private employments; ANC, antenatal care; SVD, spontaneous vaginal delivery; COR, crude odds ratio.

### Factors associated with early newborn bathing

The study showed that mothers who attained higher or more education were less likely to practice early newborn bathing compared to those who did not have formal education [adjusted odds ratio (AOR) = 0.21; 95% CI 0.06–0.66]. Mothers who resided in urban areas were less likely to practice early newborn bathing compared to mothers who resided in rural areas (AOR = 0.19; 95% CI 0.09–0.42). Mothers who gave birth using operational delivery (e.g. caesarean section and instrumental delivery) were less likely to practice early newborn bathing as compared to those who gave birth using spontaneous vaginal delivery (SVD) (AOR = 0.01; 95% CI 0.01–0.04) (Table 4).

## DISCUSSION

We investigated the prevalence of early newborn bathing and its associated factors among mothers in the Afar Region of Ethiopia. The study showed that the overall prevalence of early newborn bathing among postpartum mothers was unacceptably high to improve the health status and survival of newborns in the region. Educated mothers, those who resided in urban areas and mothers who gave birth using operational delivery were less likely to practice early newborn bathing.

Evidence shows that suboptimal newborn care practices continued to contribute to early newborn deaths in lower and middle-income countries (including Ethiopia) [25] and neonatal mortality attributed to 43% of all under-5 deaths in Ethiopia [6]. Consistent with this fact, this study showed that the practice of early newborn bathing in the Awsi Rasu Zone of the Afar Region is higher compared to findings from previously published studies in Harar (35.4%) [18] and Jimma (32.5%) [19] in Ethiopia. The potential explanation for the current finding could be that the cultural belief and social norms of the community in the Afar Region considering newborn babies as dirty when they came out of their mother’s womb may have contributed to the early bathing of the baby [26]. Abdu, *et al.* [26] supported this finding that almost half of the healthcare workers (midwives and nurses) in the Afar Region did not have adequate knowledge of appropriate newborn care practices. These findings suggest the need for interventions on essential newborn care practices to improve the awareness and practices of mothers and health care professionals in the Afar Region.

Consistent with previously published studies in Jimma [19] and Harar [18] Ethiopia, educated mothers were less likely to practice early newborn bathing. Education can improve the newborn practice of mothers in three ways. Firstly, education improves the health care-seeking behaviours of mothers, and this allows receiving information on appropriate newborn care practices [27, 28]. Secondly, a mother’s education improves the decision-making power in households, particularly in resisting the pressure from grandparents for traditional health practices [28, 29]. Finally, education offers employment opportunities for the mother, and this may create a chance to have information from their working places on appropriate newborn care practices [28]. Promoting women’s universal education can be a viable solution in improving the health and survival status of mothers and children in the Afar Region as well as in Ethiopia.

Our findings indicated that operational delivery (e.g. caesarean section and instrumental delivery) was protective against early newborn bathing, consistent with a previous study from Jimma, in Ethiopia [19]. The inverse relationship between early newborn bathing and operational delivery could be explained by that mother who gave birth at the hospital stayed more than 24 h in health facilities compared to those who gave birth through SVD, which are discharged within 6 h of birth. Residing in urban areas was also protective against the early newborn bathing practices of mothers, in agreement with similar studies conducted in Benishangul and Tigray regions in Ethiopia [30, 31]. This might be due to good access and utilization of health services in urban areas, and rural areas mothers' low educational status, and poor access and availability to infrastructures like mass media. This finding suggests the need for interventions for mothers who resided in rural areas.

This study has limitations. First, the cross-sectional nature of the study design makes it difficult to confer the direction of causality, nevertheless, the findings are consistent with other studies conducted in Ethiopia [18, 19]. Second, a recall bias in remembering some of the variables might be a possible limitation of the study. However, we restricted the current study to postpartum mothers in the first year of childbirth. Despite the limitations, the study was the first to be conducted on early newborn bathing in the pastoral community, which would support the policymakers and practitioners working in these areas.

## CONCLUSION

The practice of early newborn bathing was unacceptably high in pastoral communities of the Afar Region. Educated mothers, those who resided in urban areas and mothers who gave birth using operational delivery were less likely to practice early newborn bathing. There is a need for interventions specifically focusing on uneducated and rural mothers as part of the implementation to improve the essential newborn care practice of mothers in pastoral communities. The higher prevalence of early newborn bathing warrants further qualitative investigation of early newborn bathing using the mother’s own words.

## Data Availability

The datasets used and/or analysed during the current study available from the corresponding author on reasonable request.
